# Low anterior resection syndrome

**DOI:** 10.1002/ags3.12695

**Published:** 2023-05-23

**Authors:** Seung‐Bum Ryoo

**Affiliations:** ^1^ Division of Colorectal Surgery, Department of Surgery Seoul National University Hospital, Seoul National University College of Medicine Seoul Korea; ^2^ Colorectal Cancer Center Seoul National University Cancer Hospital Seoul Korea

**Keywords:** compliance, defecatory function, low anterior resection syndrome, quality of life, rectal cancer

## Abstract

Low anterior resection syndrome (LARS) is the distressful defecatory functional problem after sphincter‐saving surgery for rectal cancer. Although the symptoms of fecal urgency, frequency, and incontinence may develop in most of the patients after surgery, there is no definitive treatments for LARS. Multifactorial etiologies and various risk factors have been identified, but the reduction of storage capacity in the rectum is one of the main reasons for LARS. Anal sphincter muscle or nerve damage during rectoanal resection or anastomosis construction, and intersphincteric resection for low‐lying tumors or hand‐sewing anastomosis, are the absolute risk factors for LARS. Preoperative radiotherapy, postoperative complications, such as anastomosis leakage, or longer duration of stoma, are also risk factors. The severity of LARS can be confirmed using the LARS score questionnaire. The questionnaire has been translated to numerous language versions including Korean and have been validated. Diverse empirical treatments, such as loperamide, fiber, probiotics, or enema, have been tried, but the safety and efficacy have not been verified yet. The 5‐Hydroxytryptamine (5‐HT) receptor antagonist, ramosetron, used for diarrhea‐dominant irritable bowel syndrome, is one potential drug for relieving the symptoms of major LARS. A randomized‐controlled trial suggested the use of ramosetron could be safe and efficacious for patients who have major LARS after sphincter‐saving rectal cancer surgery. Novel techniques or drugs for relieving the symptoms of LARS should be developed more and further studies are necessary.

## Abstract 2

LARS can be frustrating not only in patients but also surgeons. Various etiologies and risk factors could affect the severity of symptoms, but there are no specific treatments to cure the LARS. Novel techniques or drugs for relieving the symptoms of LARS should be developed more to help the numerous patients who suffer from the distressful defectory problems after rectal cancer surgery.

## 1 INTRODUCTION

Low anterior resection syndrome (LARS) is the functional problem after sphincter‐saving surgery for rectal cancer. Despite advancements in surgical technique, chemotherapy, and radiotherapy that have increased long‐term survival, many patients who underwent surgery for rectal cancer continue to experience alterations in their bowel habits. Although these symptoms may partially subside one and a half years following surgery, the majority of individuals may experience fecal urgency, frequency, and incontinence for the rest of their lives. These functional defecation issues are the most upsetting and distressing in their lives and have a significant impact on the patients' quality of life. However, there is currently no well‐established treatment for LARS, and only symptom‐based empirical care is being used.^1^, ^2^, ^3^, ^4^

## 2 ETIOLOGIES AND RISK FACTORS

LARS has multiple etiologies and various risk factors have been identified. A critical cause of LARS is injury to the anal sphincter muscle or nerve during rectoanal resection or anastomosis construction, and intersphincteric resection for low‐lying tumors or hand‐sewing anastomosis have also been revealed as important risk factors.^5^, ^6^, ^7^ The preoperative LARS score (POLARS) is an online tool to predict bowel dysfunction severity prior to low anterior resection using a nomogram.^8^ Age, gender, total or partial mesorectal excision, tumor height, stoma, and preoperative radiotherapy are used for predictive factors in the POLARS nomogram. This POLARS can be used to help patients understand their risk of bowel dysfunction and to preoperatively highlight patients who may require additional postoperative support. But it could not consider the postoperative factors, such as complications or longer duration of stomas.^9^, ^10^ Longer periods of a defunctioning ileostomy are also thought to be risk factors, as well as neoadjuvant or adjuvant chemoradiotherapy.^11^, ^12^ Many studies revealed that anastomosis leakage and pelvic sepsis could exacerbate the symptoms of LARS because not only of aggravation of anastomotic stricture with fibrosis of surrounding tissue, but also pudendal neuropathy with lumbosacral plexopathy.^13^, ^14^

The reduction of storage capacity in the rectum also contributes to LARS. The rectum serves as a temporary reservoir for feces. Rectal compliance, which is larger than the colon, can cause the stool to be kept in the rectum until some capacity of volume increasing without pressure elevating.^15^ However, it has been reported that the neorectum, which is created with the distal colon after the rectum is removed, exhibits hyperactive motility, and altering this rectal physiology of motility could eventually result in increased stool frequency and urgency.^16^, ^17^ Denervation of the autonomic extrinsic nerve may affect colonic motility by increasing smooth muscle contractions or by causing defecatory disorders due to improper evacuation.^18^, ^19^ However, even without extrinsic innervation regulations, the intrinsic enteric nervous system (ENS) can create colonic peristalsis. Because the colon moves feces from the proximal to the distal section as soon as they enter the lumen, it has been considered that colonic patterns of contraction and relaxation cannot take the place of the rectum's storage function.^20^, ^21^

## 3 LOW ANTERIOR RESECTION SYNDRIME SCORE

The LARS score questionnaire can determine the severity of symptoms of LARS. Five questions have been included, which are incontinence for flatus or liquid stools, frequency of bowel movements, clustering of stools, and urgency. The major LARS can be determined if the score is higher than 30, and minor LARS can be defined if the score is higher than 20.^22^, ^23^ The reported prevalence of major LARS is about 40%–60%.^24^, ^25^ The LARS score questionnaire has been translated to numerous language versions, including Korean, and have been validated.^26^, ^27^ The symptoms can be classified with two dominant patterns, which consists of incontinence‐dominant or frequency‐dominant LARS from our previous study.^28^ There might be different risk factors for LARS in two different patterns. The incontinence‐dominant pattern is related to preoperative radiotherapy and postoperative complications, whereas the frequency‐dominant pattern is related to low tumor location. Frequency‐dominant LARS had more profound associations with poor quality of life. We can use these patterns for preoperative prediction and more specific treatment of LARS can be provided from further studies, according to the dominant symptom patterns.

## 4 TREATMENT OF LOW ANTERIOR RESECTION SYNDROME

Numerous techniques have been trying to improve the symptoms of LARS, but there is no specific treatment proven for LARS. The various treatment modalities for LARS are described in Table 1. It has been considered that each treatment may have advantages and disadvantages in clinical practice and have been usually selected without strong evidence.

**TABLE 1:** Treatments of low anterior resection syndrome

Treatments	*Safety	**Efficacy	Evidence	Clinical practice
Fiber	+	±	Empirical [2]	Can be tried, but rarely effective
Probiotics	+	−	RCTs [30, 31]	Not usually used
Loperamide	±	++	Empirical [2]	The most commonly prescribed, But has some safety issues
5HT receptor antagonist	+	+	RCT [41] case‐series [40]	Emerging, But needs more studies
Pelvic floor muscle training	+	±	RCTs [43, 44] systematic reviews [42]	Controversies for outcomes Long‐term effects have not been proven
Sacral nerve stimulation	±	+	Prospective studies [47, 48] systematic review [49]	Mechanism of action has not been verified Long‐term effects have not been proven
Transanal irrigation	±	++	RCT [46] systematic review [45]	Should be performed carefully
Permanent stoma	+	+++	Prospective study [56] case‐series [57]	Lower quality of life

*Note*: *Safety, +, safe, ±, may not be safe in some cases. **Efficacy, +++, absolutely efficient, ++, efficient, +, relatively efficient, ±, efficient in selective cases, −, not efficient.

Abbreviation: RCT, randomized controlled trial.

### 4.1 Diet and lifestyle modification

There has been no evidence of diet and lifestyle modification improvement in the treatment for LARS. For the symptomatic management of LARS, fiber with laxatives or enema may be tried, but the effects have not been proven.^2^ There have been no studies with a high level of evidence in the high‐ or low‐fiber diet for LARS treatment.

### 4.2 Medications

Similar to fecal incontinence, many empirical treatments are employed. Loperamide, an agonist of the μ‐opioid receptor, is the most commonly used drug. Because it slows down intestinal contractions and propagations, it may have an effect on diarrhea or fecal incontinence. However, it should only be administered to limited cases and temporally, as severe constipation or toxic colitis are one of the possible side effects.^2^, ^29^ Probiotics could also prevent diarrhea, but the results have not been satisfactory in LARS yet.^30^ VSL#3 was tried to improve postoperative bowel function in a randomized clinical trial, but has not been verified, with no statistically significant difference between the treatment and placebo control.^31^

The 5‐Hydroxytryptamine (5‐HT) receptor antagonist has been tried for relieving the symptoms of major LARS.^32^ It also has been verified to be closely associated with functional gastrointestinal (GI) disorders, such as irritable bowel syndrome (IBS).^33^ The plasma level of 5‐HT can be increased in IBS with diarrhea (IBS‐D) and decreased in IBS with constipation (IBS‐C). The patients who have IBS‐D could present visceral hypersensitivity and increased bowel motility against stimulations with psychological stress. Recently, 5‐HT_3_ receptor antagonists can be used for the treatment of IBS‐D, and has been revealed slow bowel movement and improve stool consistency and urgency.^34^ Ramosetron is one of the 5‐HT_3_ receptor antagonists, which have higher potency of affinity and selectivity compared to other drugs.^35^ Many clinical trials also have proved the efficacy of ramosetron for relieving the symptoms of IBS‐D and the safety of potential side effects is only about 7% of constipation or defecation difficulty.^36^, ^37^, ^38^, ^39^ Because of the similarity of symptoms between LARS and IBS‐D, 5‐HT_3_ receptor antagonists was considered in use for the treatment of LARS in a pilot study.^40^ The incontinency score, urgency grade, and number of defecations per day were significantly improved after 1 month of ramosetron treatment. The effect seemed to be more prominent in patients who had a lower anastomosis line and were treated within 6 mo after the sphincter‐saving surgery. Although this study included a small number of patients and did not use a control group, the results showed the possibility of using 5‐HT receptor antagonists to manage severe symptoms of LARS, which seemed to have a similar hypermotility pathophysiology as IBS‐D. We performed a randomized controlled trial to assess the efficacy and safety of ramosetron (Irribow^®^), a 5‐HT_3_ receptor antagonists, for the treatment of LARS.^41^ This randomized controlled trial in male patients who underwent sphincter‐saving surgery for rectal cancer demonstrated the efficacy and safety of the 5‐HT_3_ receptor antagonist, using ramosetron for the treatment of LARS. The LARS score significantly decreased after 4 weeks of ramosetron treatment and significantly lower than conservative treatment. The proportions of major LARS after 4 weeks were 58.3% (*n* = 28/48) in the ramosetron group versus 82.0% (*n* = 41/50) in the control group, with the difference of 23.7% being statistical significance (95% confidence interval [CI] = 5.58 ~ 39.98%, *P* = 0.011). The quality of life after 4 weeks was significantly better in the ramosetron group. Two patients (4.2%) experienced hard stool in the ramosetron group, but there were no serious adverse events related to the treatment during the study periods.

### 4.3 Pelvic floor rehabilitation

It has been proposed that pelvic floor rehabilitation can reduce LARS symptoms, and in a study of a literature review, most of the studies presented better functional outcomes after pelvic floor rehabilitation.^42^ We also performed a prospective study for verifying the effects of anal sphincter exercise, but it is still unclear how the biofeedback training differs from self‐training with how the Kegel maneuver differs.^43^ A recent randomized trial investigated the effectiveness of the pelvic floor muscle training after rectal cancer surgery, but they revealed lower proportions and faster recovery of bowel symptoms in the pelvic floor muscle training group only up to 6 mo, and the effects did not last longer at 12 months.^44^

### 4.4 Transanal irrigation

Transanal irrigation or enema on the patient's own for emptying the neorectum with or without washout can provide pseudo‐continence and timed‐defecation. It can prevent fecal leaks or the nighttime bowel movement, and improve the quality of life.^45^ However, it should be performed very carefully because of the risk of perforation and anastomosis leakage, and it should not be recommended during the early postoperative periods following rectal cancer surgery. A multicenter randomized trial to evaluate the transanal irrigation as a treatment for LARS confirmed that it reduces symptoms in LARS and improves the quality of life.^46^ However, the study was performed with a small number of participants and could involve some potential complications such as pain at irrigation, difficulties inflating water with keeping the catheter in place, or bowel emptying between irrigations in that study. More studies are still needed to verify the safety and efficacy in transanal irrigation.

### 4.5 Surgical treatments

Sacral nerve stimulation (SNS) has been reported to improve LARS.^47^, ^48^ However, several studies found that the symptoms that improved after SNS were clustering or urgency rather than incontinence or frequency, and the mechanism underlying the change is still unknown.^49^, ^50^ Specific surgical techniques have been tried to prevent LARS. The reservoir volume of the neorectum can be increased with formation of a colonic J pouch side‐to‐end coloanal anastomosis and these procedures have been presented with better functional outcomes than straight coloanal anastomosis for the first year after surgery. But it seemed not to continue for a long period, and the improvement of symptoms could be similar after about one or 2 y.^51^, ^52^ It can be assumed that the capacity of the reservoir function of the neorectum can increase to some degree over time, even in the straight coloanal anastomosis.^11^, ^53^ Recently, minimally invasive surgery has been proven to have benefits for short‐term outcomes of early postoperative recovery with long‐term oncologic safety. However, in terms of functional outcomes related to LARS, the incidence is still high. Studies have been reported that laparoscopic or robotic surgery for rectal cancer had more than 60%–70% of LARS, and transanal total mesorectal excision presented similar comparative outcomes.^54^, ^55^ Finally, permanent stoma might be inevitable after failure of every effort, and could improve the quality of life.^56^, ^57^ Sometimes patients who suffer from the severe symptoms of LARS strongly ask the surgeon to make the permanent stoma first. The specific care for stoma can have advantages for hygienic problems and be preferred to a devastating daily life from the frequent and urgent bowel movements or fecal incontinence with painful perianal skin irritation.

## 5 CONCLUSION

LARS can be frustrating, not only for the patients but also the colorectal surgeons. Various etiologies and risk factors could affect the severity of symptoms, but there are no specific treatments to cure the LARS. Novel techniques or drugs for relieving the symptoms of LARS should be developed to help the numerous patients who suffer from the distressful defecatory problems after rectal cancer surgery.

## FUNDING INFORMATION

This study was supported by a grant of Basic Science Research Program through the National Research Foundation (NRF) of Korea, funded by the Ministry of Sciences & ICT, Republic of Korea (grant number: 2019R1C1C1006654).

## CONFLICT OF INTEREST

The author declare no conflicts of interests for this article.

## ETHICS STATEMENTS

Approval of the research protocol: N/A.

Informed consent: N/A.

Registry and the Registration No. of the study/trial: N/A.

Animal studies: N/A.
